# 

**Published:** 1875-02

**Authors:** 


On the Treatment of Pleurisy: with an appendix of cases, showing the value of combinations of croton oil, ether, and iodine, as counter-irritants in other diseases. By John W. Corson, M.D., etc. New York: William Wood & Co., No. 27 Great Jones street. 1874. 12 mo. cloth, pp. 31. Price 50c.

---

A Series of American Clinical Lectures. Edited by E. C. Seguin, M.D. Volume 1, No. 1. On Diseases of the Hip Joint. By Lewis A. Sayre, M.D., Professor of Orthopedic Surgery and Clinical Surgery in Bellevue Hospital Medical College, N. Y. New York: G. P. Putnam's Sons. 1875. Octavo, paper, pp. 24. Price 40c.

---

A Sketch of the Early History of Practical Anatomy. The introductory address to the course of lectures on anatomy at the Philadelphia School of Anatomy. By William W. Keen, M.D., Lecturer on Anatomy and Operative Surgery in the Philadelphia School of Anatomy, etc. Philadelphia: J. B. Lippincott & Co. 1874. 12 mo., cloth, pp. 43. Sold in Atlanta by S. P. Richards & Son.

---

Migrants and Sailors considered in their relation to the Public Health. A.—Some defects in the Immigration Service affecting the Sanitary interests of the country. By John M. Woodworth, M.D., Supervising Surgeon U. S. Marine Hospital Service. B.—Sailors as Propagators of Disease. By Heber Smith, M.D., Surgeon U. S. Marine Hospital Service. Cambridge: Riverside Press. 1875.

LETTER TO A COMMITTEE OF CITIZENS on the proposed Schuylkill Drove-yard and Abattoir. By John H. Rauch, M.D., Treasurer of the American Public Health Association, etc. With medical opinions on the subject. Philadelphia: Collins. 1874. pamphlet, pp. 15.

---

IN THE COURT OF COMMON PLEAS of Philadelphia County. In Equity. Sellers et. al. vs. the Pennsylvania Railroad Company et al. Complainants' affidavits on motion for injunction to restrain erection of slaughter-house. 1874.

---

ON REFLEX IRRITATIONS throughout the Genito-Urinary Tract, resulting from contraction of the urethra at or near the meatus urinarius, congenital or acquired. By Fessenden N. Otis, M.D., Clinical Professor of Genito-Urinary Diseases in the College of Physicians and Surgeons, N. Y. Read before the New York Academy of Medicine. New York: McDivitt, Campbell & Co. 1875. Pamphlet, pp. 37.

---

REPORT OF THE SURGIAL CASES treated in the St. John's Riverside Hospital, Yonkers, N. Y., during the year 1873. By J. H. Pooley, M.D., Surgeon to the Hospital, etc. New York: D. Appleton & Co. 1875. Pamphlet, pp. 29.

---

NEAR-SIGHT TREATED BY ATROPIA. With Tables. By Hasket Derby, M.D. Surgeon to the Massachusetts Charitable Eye and Ear Infirmary, at Boston. New York. 1875. Pamphlet, pp. 8.

---

PROCEEDINGS of the first Annual Meeting of the Eastern Medical Association, at Newbern, N. C., November 3, 1874. Pamphlet, pp. 71.

---

CHOMEL says: "The proper aim of the physician is, first, not to do harm, and second, to try to do good. The physician is not to treat diseases, but patients affected with diseases." Bordeau says: "I was dogmatic at twenty, an observer at thirty, an empiric at forty, and now, at fifty, I have no longer any system."

These maxims are as true and as important to-day as when they were first uttered. Yet, how many of us remember and act in accordance with their teaching?—*Detroit Review of Medicine and Pharmacy.*

